# Child Protection System Interactions for Children With Positive Urine Screens for Illicit Drugs

**DOI:** 10.1001/jamanetworkopen.2024.3133

**Published:** 2024-03-21

**Authors:** Rebecca Rebbe, Denise Malicki, Nadia Siddiqi, Jeannie S. Huang, Emily Putnam-Hornstein, Natalie Laub

**Affiliations:** 1 University of North Carolina at Chapel Hill School of Social Work, Chapel Hill; 2 University of California, San Diego; 3 Rady Children’s Hospital, San Diego, California; 4 Chadwick Center for Children and Families, San Diego, California; 5 Nova Southeastern University, Fort Lauderdale, Florida

## Abstract

**Importance:**

Young children are ingesting illicit drugs at increased rates, but it is unknown what the associated child protection system (CPS) responses are when a child tests positive.

**Objective:**

To document the child protection system involvement and the characteristics of children who test positive for illicit substances.

**Design, Setting, and Participants:**

This retrospective cross-sectional study linked medical discharge and child protection system administrative data. The setting was Rady Children’s Hospital San Diego, a free-standing pediatric hospital in California. Participants included all emergency department and inpatient medical encounters involving children aged 12 years or younger with a positive urine drug test between 2016 and 2021. Statistical analysis was performed from February 2023 to January 2024.

**Exposure:**

Drug type, including amphetamines, barbiturates, benzodiazepines, cannabis, cocaine, fentanyl, opiates, and phencyclidine.

**Main Measures and Outcomes:**

CPS responses associated with the medical encounter including reports, substantiations, case openings, and out-of-home placements.

**Results:**

A total of 511 emergency department and inpatient medical encounters involving children had a positive drug test (262 [51.3%] were female; 309 [60.5%] were age 6 years or younger; fewer than 10 [<3.0%] were American Indian or Alaska Native; 252 [49.3%] were Hispanic [any race], 20 [3.9%] were non-Hispanic Asian, 56 [11.0%] were non-Hispanic Black, 143 [28.0%] were non-Hispanic White, 36 [7.0%] had other or unknown race and ethnicity; 233 [43.6%] had a CPS report prior to the medical encounter). Following the positive screen, 244 (47.7%) were reported to child protection, and 61 (11.9%) were placed out-of-home within 30 days. Mean (SD) quarterly counts of encounters with positive drug tests doubled after the COVID-19 pandemic onset (32.9 [9.8]) compared with prior to the pandemic onset (16.5 [4.7]); for encounters positive for cannabis, mean (SD) quarterly counts were 3 times as high after the pandemic onset than prior (16.6 [4.7] vs 5.7 [2.9]). Encounters for children under age 1 were significantly more likely to have associated child protection reports (relative risk [RR], 2.91 [95% CI, 2.21-3.83]) and child protection case openings (RR, 1.71 [95% CI, 1.07-2.72]) than encounters involving older children.

**Conclusions and Relevance:**

In this cross-sectional study of emergency department and inpatient medical encounters, less than half of children with positive urine drug screens were reported to CPS; out-of-home placements were uncommon. With increased encounters for positive drug tests, it is unclear what services these children and families are receiving.

## Key Points

**Question:**

What are the child protection system (CPS) interactions for children aged 12 years or younger who test positive for illicit drugs during emergency department and inpatient medical encounters?

**Findings:**

In this cross-sectional study of 511 emergency department and inpatient medical encounters involving a child with a positive drug screen, 47.7% were reported to CPS, and 11.9% resulted in out-of-home placement within 30 days; 43.6% of the children had a prior CPS report for concerns of child maltreatment.

**Meaning:**

The findings of this study suggest that fewer than half of encounters for children with positive drug screens result in reports to CPS and that out-of-home placements are uncommon.

## Teaser

This cross-sectional study investigates the child protection system (CPS) involvement and the characteristics of children who test positive for illicit substances.

## Introduction

The use of substances such as cannabis and opioids in adults is on the rise in the United States.^1,2,3,4,5,6,7,8,9,10,11,12^ Children who reside in homes where adult drug use is occurring are at risk of accidentally ingesting those substances.^13,14,15^ Recent research indicates that there has been a 26% increase in illicit drug ingestions in children since the onset of COVID-19 and pediatric hospitalizations for cannabis poisoning has doubled in areas with recreational legalization.^16,17,18^ Young children often ingest these drugs through exploratory means as they are unaware of the dangers; caregivers who are using substances may be less able to monitor their children’s behavior.^19,20^

When a young child tests positive for an illicit drug, a report to the child protection system (CPS) may occur, however reporting is variable depending on geographic location, institutional practice, state laws, and may be influenced by bias.^21,22,23,24^ Prior research suggests that parental and/or caregiver drug use is a common concern on CPS reports, CPS investigator assessments, and out-of-home placements; since 2008, the number of entries into foster care due to parental substance use has risen and 23% of removals are directly attributed to parental drug use.^25,26^ Prior research has primarily focused on CPS interactions that follow prenatal substance exposure, finding that around half of all infants diagnosed with substance exposure during pregnancy are reported to CPS, but that these interactions vary by substance type.^27,28,29^ A previous study that examined drug exposure beyond the neonatal period using hospital data from 2006 to 2008 found that only 4% of cases were reported to CPS and reporting rates varied by substance type.^30^ Given increases in child ingestion rates, and the legalization of cannabis in many states, understanding both reporting practices and CPS responses by drug type when a child tests positive is important.^17,18,31,32,33,34^

In the present study, we use administrative data from a large free-standing children’s hospital linked to statewide CPS records to (1) document the characteristics of children who test positive for illicit drugs in emergency department (ED) and inpatient medical settings; and (2) examine associated CPS interactions (reports, substantiation, case opening, and out-of-home placement) by substance type.

## Methods

### Study Population and Setting

**Figure 1.**
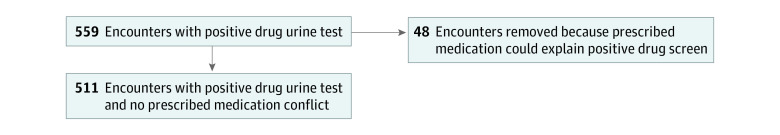
Flow Diagram of the Study Population Selection

In this cross-sectional study, we used ED and inpatient medical encounter records from a tertiary children’s hospital. We selected all medical encounters for children who were at least 28 days of age through 12 years of age with a positive urine drug screen between January 1, 2016, and December 15, 2021 (n = 559). We excluded children under 28 days of age as our study was not focused on prenatal exposure. Rather, we sought to examine children who had a positive drug screen that was not due to maternal drug use during pregnancy or at delivery. Age 12 years was the upper cutoff based on previous research in an attempt to distinguish between unintentional and intentional ingestion in children.^35^ For encounters where amphetamines, opiates, benzodiazepines, and barbiturates were the only positive drug test, we examined the medication prescribed during the medical encounter and removed encounters (n = 48) that included prescribed medications which could cause the positive drug test. The final study population was 511 medical encounters which represented 489 unique children (Figure 1).

Urine drug screens are performed at this children’s hospital as standard of care when a child presents with a toxidrome concerning for ingestion, poorly explained altered mental status, or brought in by law enforcement or CPS because drugs were found in the home. eTable 1 in Supplement 1 presents the frequency and description of the most common diagnoses for the study population. Medical records were probabilistically linked to statewide CPS records at the child-level for January 2010 through March 31, 2022, using personally identifiable information and methods detailed elsewhere.^36,37^ This study had institutional review board approvals from the California Committee for the Protection of Human Subjects, University of Southern California, and University of California San Diego; and was granted a waiver of participant consent because we used administrative data and the research team had no contact with participants. We followed the Strengthening the Reporting of Observational Studies in Epidemiology (STROBE) reporting guideline.

### Measures and Outcomes

We coded each encounter based on the drug type that was positive in the screen: amphetamines and/or methamphetamine, cannabis, cocaine, fentanyl, opiates, benzodiazepines, barbiturates, and phencyclidine (PCP). Both fentanyl (a synthetic laboratory-made opioid) and the naturally derived opiate drugs (heroin, morphine, codeine) are included in screening protocols. Fentanyl was added to the standard urine drug screen in October 2020, whereas the remainder of the drugs have been included in the urine drug screen since 2010. As some children tested positive for multiple drugs (n = 43), we included any drug with a positive test and a binary indicator for those children who tested positive for multiple drugs. A binary variable documented the highest level of care the child received during the medical encounter: ED or inpatient.

Five binary variables documented each child’s interactions with CPS: (1) a CPS report of alleged maltreatment more than 2 days prior to the medical encounter admission date; (2) a CPS report within the medical encounter window of 2 days before admission through 2 days after discharge to accommodate minor data entry errors; (3) a CPS report within the encounter window was substantiated; (4) a CPS case opened for services following the medical encounter window (regardless of placement decisions) and within 45 days, the timeframe for CPS investigations in this jurisdiction (for encounters where there was a case already open at the time of admission, these were included as case opened [n = 16]); and (5) a CPS placement episode that began within 30 days of the medical encounter discharge date (we examined a longer period, 60 days, and the number of placements was the same). CPS reports were counted regardless of whether they were screened-in for investigation.

Medical encounter records were used to code the child’s age at the time of admission (<1 year, 1-6 years, 7-12 years), child sex (male or female), whether the encounter was paid for using public health insurance (yes or no), and child race and ethnicity (any race Hispanic, American Indian or Alaska Native, non-Hispanic Asian/Pacific Islander, non-Hispanic Black, non-Hispanic White, other/unknown race (includes selections for “Other” and “Decline to Answer”). Race and ethnicity were assessed in this study due to concerns of the role of bias with CPS reporting.^24^ Additionally, because the study period included encounters before and during the COVID-19 pandemic, we included a binary variable indicating whether the medical encounter started after the COVID-19 pandemic onset, defined as March 16, 2020, as has been done in previous studies.^38,39^

### Statistical Analysis

Frequencies were calculated to characterize the study population. We tallied the number of medical encounters with a positive drug screen by quarter (3 months) and substance type, plotting these counts over time. We stratified our frequency counts by child characteristics and CPS report status, and we used χ^2^ tests to assess differences in the distributions. We also calculated the percentage of encounters for each positive drug category with subsequent CPS interactions.

We then ran 2 regression models which included the substance type and controlled for child and encounter characteristics (age, sex, race and ethnicity, public health insurance, encounter setting, COVID-19 onset) as these variables may confound the outcomes. We used modified Poisson regression with a robust error variance (sandwich estimation) to calculate the relative risks given that odds ratios can overestimate the effect sizes for common (>10%) outcomes.^40,41^ The first model estimated the risk of a CPS report during the medical encounter associated with the positive drug test. The second model estimated, for the subset of encounters reported to CPS, the risk of CPS opening a case for services beyond the investigation. Results are presented as relative risks (RRs) with 95% CI. All analyses were performed from February 2023 to January 2024 using R version 4.1.1 (R Project for Statistical Computing). An a priori 2-sided *P* < .05 was considered statistically significant.

## Results

There were 511 medical encounters with a positive drug screen during the study time frame; 262 (51.3%) were female, and 309 (60.5%) were aged 6 years or younger, with 59 (11.5%) under 1 year of age; fewer than 10 (<3.0%) were American Indian or Alaska Native; 252 (49.3%) were Hispanic (any race), 20 (3.9%) were non-Hispanic Asian, 56 (11.0%) were non-Hispanic Black, 143 (28.0%) were non-Hispanic White, and 36 (7.0%) had other or unknown race and ethnicity. The Table presents the population characteristics stratified by whether a CPS report was made during the medical encounter. Cannabis (213 encounters [41.7%]) was the most frequent illicit substance identified in the urinalysis tests, followed by benzodiazepines (139 encounters [27.2%]), amphetamines (114 encounters [22.3%]), opiates (38 encounters [7.4%]), and fentanyl (36 encounters [7.0%]). More than half the medical encounters were inpatient admissions (288 encounters [56.4%]), two-thirds were paid for using public health insurance (342 encounters [66.9%]), and for 233 encounters (43.6%) the child had a previous report to CPS. Statistically significant differences in the distributions of encounters by CPS report status were observed by positive drug type. Compared with encounters not reported to CPS, encounters reported to CPS more commonly had a positive test for cannabis (CPS report: 130 encounters [53.3%] vs no CPS report: 83 encounters [31.1%]) and amphetamines (CPS report: 65 encounters [26.6%] vs no CPS report: 49 encounters [18.4%]) (*P* < .001). Encounters with children under age 1 (42 encounters [17.2%]) and aged 1 to 6 years (128 encounters [52.5%]) composed higher proportions of those reported to CPS (*P* < .001). Approximately three-quarters of encounters reported to CPS were paid for using public health insurance, which was higher than those not reported to CPS (182 of 244 encounters [74.6%] vs 160 of 267 encounters [59.9%]; *P* < .001). eTable 2 in Supplement 1 presents the study population characteristics by positive drug type.

**Table.** Study Population Characteristics by CPS Report Status During Medical Encounter

	All positive drug tests (n = 511)	No CPS report (n = 267 [52.3%])	CPS report (n = 244 [47.7%])	*P* value
Drug^a^				
Cannabis	213 (41.7)	83 (31.1)	130 (53.3)	<.001
Benzodiazepines	139 (27.2)	116 (43.4)	23 (9.4)	
Amphetamine	114 (22.3)	49 (18.4)	65 (26.6)	
Opiate	38 (7.4)	24 (9.0)	14 (5.7)	
Fentanyl	36 (7.0)	(<5)	(<15)	
Barbiturates	25 (4.9)	(<10)	(<5)	
Cocaine	<10 (<3.0)	NR^b^	NR^b^	
PCP	<10 (<3.0)	NR^b^	NR^b^	
Multiple	52 (10.2)	27 (10.1)	25 (10.2)	
Encounter type				
Emergency department	223 (43.6)	113 (42.3)	110 (45.1)	.59
Inpatient	288 (56.4)	154 (57.7)	134 (54.9)	
Age category				
< 1 y	59 (11.5)	17 (6.4)	42 (17.2)	<.001
1-6 y	250 (48.9)	70 (26.2)	128 (52.5)	
7-12 y	202 (39.5)	180 67.4)	74 (30.3)	
Child sex				
Female	262 (51.3)	146 (54.7)	116 (47.5)	.13
Male	249 (48.7)	121 (45.3)	128 (52.5)	
Public health insurance				
Yes	342 (66.9)	160 (59.9)	182 (74.6)	<.001
No	169 (33.1)	107 (40.1)	62 (24.4)	
Child race/ethnicity				
American Indian/Alaska Native	<10 (<3.0)	NR^b^	NR^b^	.01
Any race Hispanic	252 (49.3)	127 (47.6)	125 (51.2)	
Non-Hispanic Asian	20 (3.9)	NR^b^	NR^b^	
Non-Hispanic Black	56 (11.0)	24 (9.0)	32 (13.1)	
Non-Hispanic White	143 (28.0)	74 (27.7)	69 (28.3)	
Other/unknown^c^	36 (7.0)	25 (9.4)	15 (6.1)	
After COVID-19 Onset?				
Yes	234 (45.8)	101 (37.8)	133 (54.5)	<.001
No	277 (54.2)	166 (62.2)	111 (45.5)	
Prior CPS report				
Yes	233 (43.6)	107 (40.1)	116 (47.5)	<.001
No	288 (56.4)	160 (59.9)	128 (52.5)	

^a^ Drug type is not mutually exclusive.

^b^ Masked due to small cell sizes.

^c^ “Other/Unknown” race and ethnicity includes “Other” race and “Decline to Answer.”

**Figure 2.**
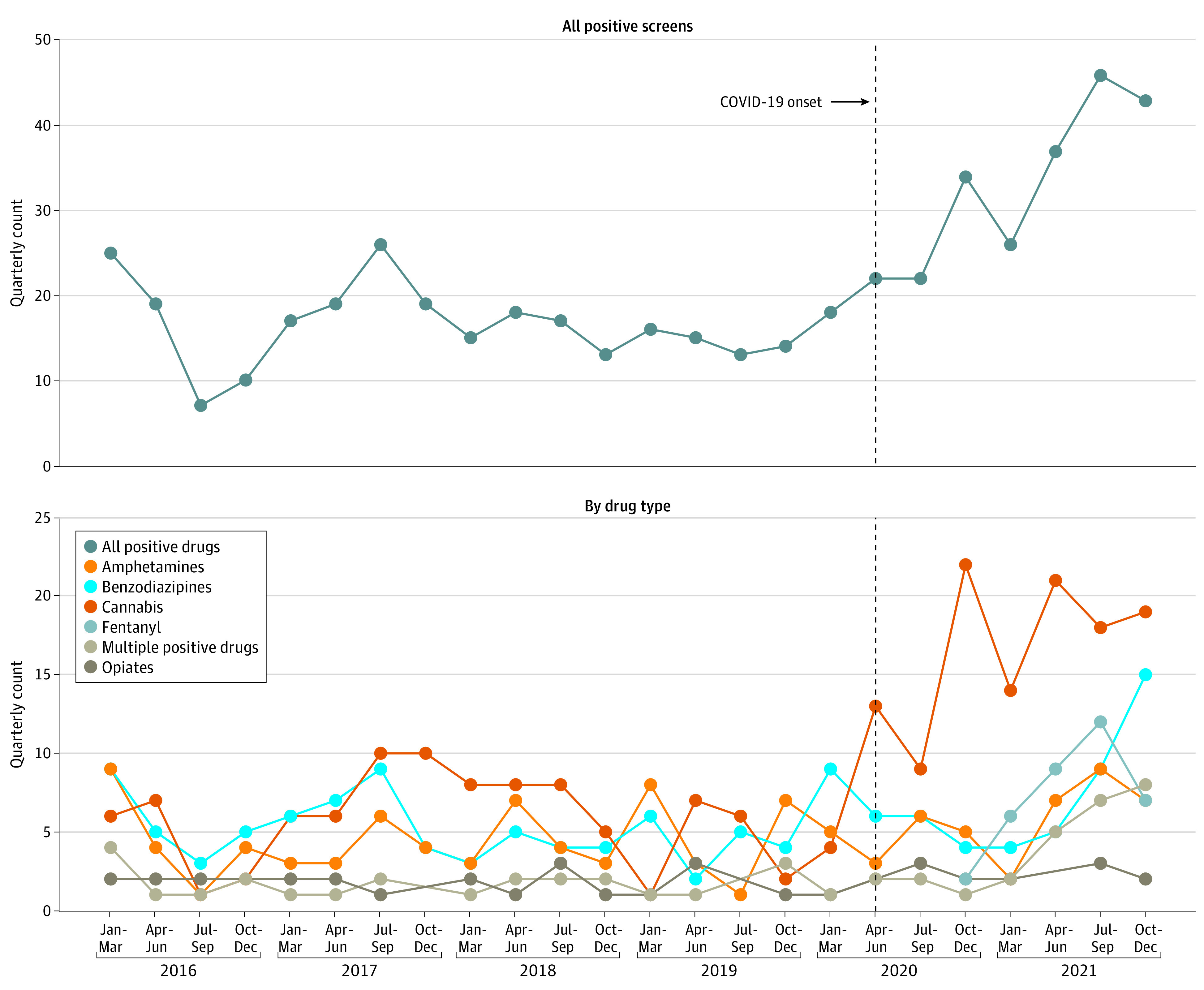
Quarterly Counts of Positive Urine Drug Tests by Drug Type

Figure 2 presents the quarterly counts of encounters with positive drug tests overall and by substance type over time. Counts of positive drug tests overall and related to cannabis, fentanyl, and multiple positive drugs increased after the onset of the COVID-19 pandemic, with an overall peak observed in the third quarter of 2021. Mean (SD) quarterly counts of all positive tests were higher after the pandemic onset (32.9 [9.8]) than prior to the pandemic onset (16.5 [4.7]), which was also true for positive cannabis tests (after pandemic onset: 16.6 [4.7]; prior to pandemic: 5.7 [2.9]).

**Figure 3.**
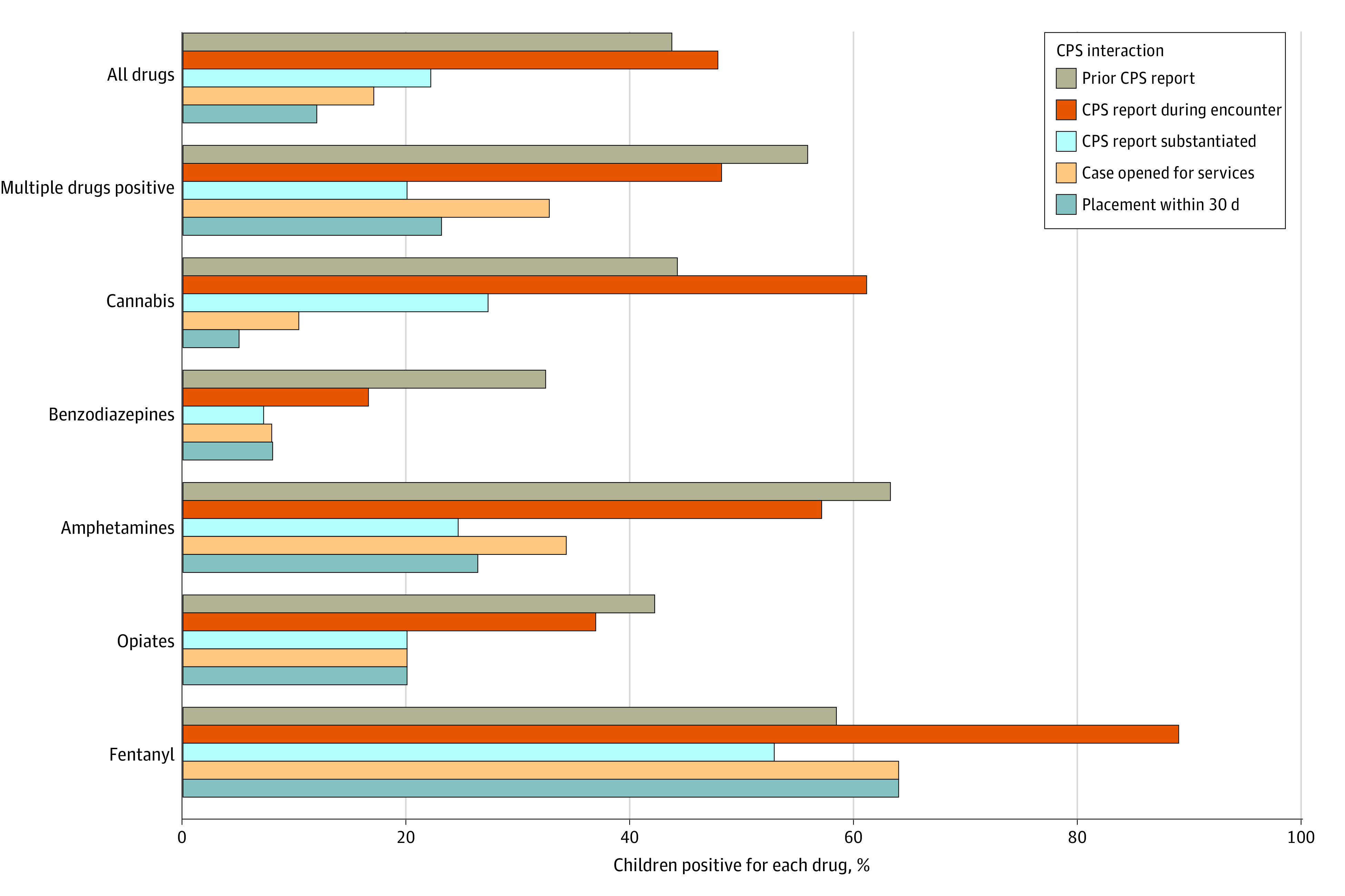
Percentage of Child Protection System (CPS) Interactions Experienced by Positive Drug Category

Figure 3 presents the percentage of encounters testing positive in each drug category who experienced the measured CPS interactions. Among all 511 children with a positive drug test, 244 (47.7%) were reported to CPS during the medical encounter, 113 (22.1%) had an allegation of maltreatment substantiated by CPS, 87 (17.0%) had a case opened for services by CPS, and 61 (11.9%) were placed out-of-home by CPS within 30 days. χ^2^ tests documented statistically significant differences in the distributions for each of the CPS interactions by drug type (*P* < .001). The highest rates of CPS interactions were for encounters with positive tests for fentanyl: 19 of 36 (52.8%) had an allegation substantiated by CPS and 23 of 36 (63.9%) had a case opened for services and were placed out-of-home within 30 days. Cases were opened for services for 17 of 52 encounters with multiple positive drugs (32.7%), 39 of 114 amphetamine encounters (34.2%), and 22 of 213 cannabis encounters (10.3%). Similarly, out-of-home placements occurred less frequently for positive screens of amphetamines (30 of 114 [26.3%]), benzodiazepines (11 of 139 [8.0%]), and cannabis (<5% of 213).

**Figure 4.**
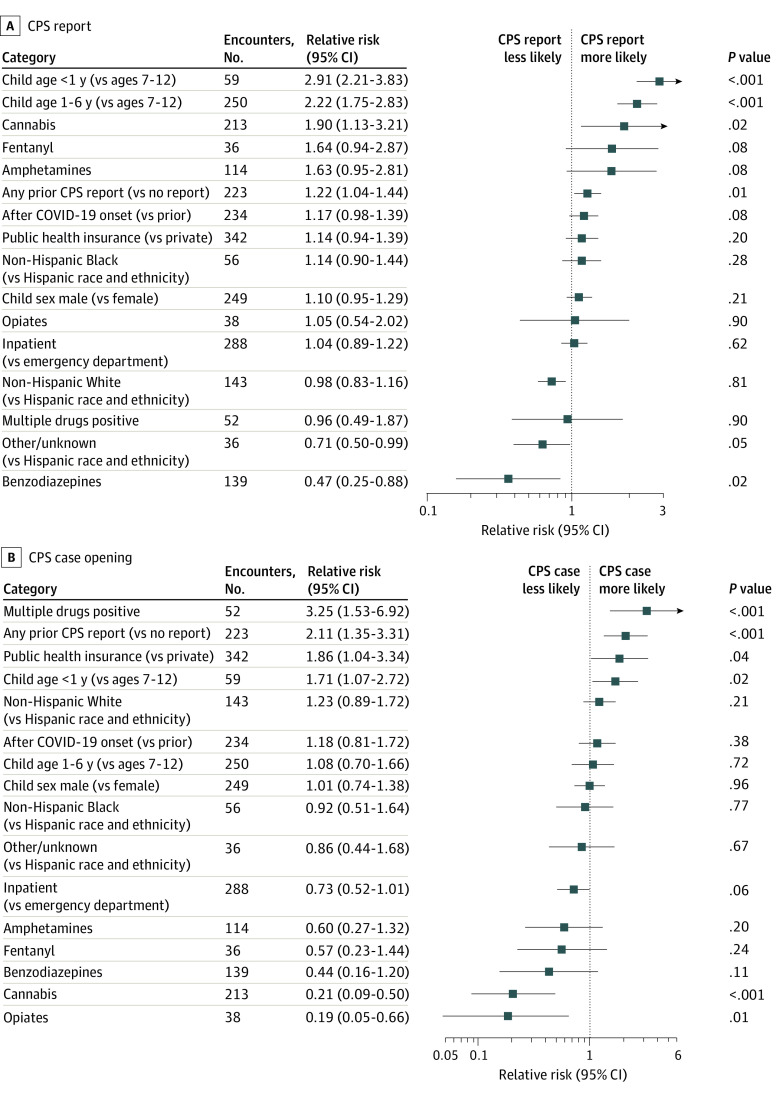
Relative Risk of Regression Models for Child Protection System (CPS) Outcomes of Report and Case Opening

Figure 4 presents encounter characteristics associated with the CPS outcomes of reports and case openings in the 2 regression models (adjusted with covariates). Child age had the highest risk of a CPS report as children under age 1 year (RR, 2.91 [95% CI, 2.21-3.83]) and aged 1 to 6 years (RR, 2.22 [95% CI, 1.75-2.83]) had higher risk of a report than children aged 7 to 12 years. For the outcome of a CPS case opened, children under age 1 year (RR, 1.71 [95% CI, 1.07-2.72]) had higher risk than children aged 7 to 12 years, but there was no difference for children aged 1 to 6 years.

The substance type also had statistically significant differences in risk for both CPS report and CPS case openings. Encounters with positive tests for cannabis had an increased risk of a CPS report (RR, 1.90 [95% CI, 1.13-3.21]) but lower risk of a case opened (RR, 0.21 [95% CI, 0.09-0.50]) than encounters not testing positive for cannabis. Encounters that included positive tests for multiple drugs had increased risk of a case opening (RR, 3.25 [95% CI, 1.53-6.92]), but not an increased risk of a CPS report compared with encounters that tested positive for a single drug type.

Encounters where the child had a prior CPS report had higher risk of a CPS report during the medical encounter (RR, 1.22 [95% CI, 1.04-1.44]) and a case opened for services (RR, 2.11 [95% CI, 1.35-3.31]) than those without a prior CPS report. Encounters paid for with public health insurance had higher risk of having a CPS case opened (RR, 1.86 [95% CI, 1.04-3.34]), but not to being reported to CPS. No statistically significant differences were observed by child race and ethnicity, child sex, or encounter type in the adjusted regression models.

## Discussion

Using linked hospital discharge and CPS administrative data, we documented that fewer than half (47.7%) of medical encounters for young children with a positive drug screen were reported to CPS at time of medical encounter, and 11.9% were placed out-of-home within 30 days. Importantly, the rates of CPS interactions differed by drug type, with deeper CPS penetration for children who tested positive for fentanyl. Our results align with previous studies regarding prenatal substance exposure, which found that those children who were reported to and placed by CPS differed by substance type.^27,28,29,42^ These findings continue to suggest that both reporting parties and CPS workers take the type of substance into account when assessing the need for CPS intervention. Similar to previous studies using linked administrative data of prenatal substance exposure and medical encounters with diagnosed child maltreatment, immediate removals by CPS were uncommon.^28,29^ This result is likely influenced by local policies, procedures, and laws regarding child maltreatment.^43,44^

Mirroring national data, our study found a sustained increase in children through 12 years of age testing positive for drugs after March 2020, particularly for cannabis and fentanyl.^18,47^ The increase of positive fentanyl tests may be associated with the increase of fentanyl use and availability in the US.^48,49,50^ The increase in cannabis ingestions is likely multifactorial and may include legalization policies, the effect of the pandemic, and product packaging and marketing, but more research is needed to understand this trend.^17,18,19,47,51,52^

This study reinforced that drug ingestions requiring medical attention are not limited to older children where intent (eg, accidental vs self-harm) may be in question. Almost two-thirds of the children in our study were 6 years of age or younger. Given the increased vulnerability of younger children, it was unsurprising that younger children were found to have higher risk of CPS reports and case openings. These findings highlight the need for public health campaigns that increase awareness and educate parents about the importance of safe storage of drugs and paraphernalia.^18^ Given that 54.0% of cannabis ingestions occurred for children between ages 1 and 6 years, this raises concerns about the attractive packaging of cannabis products and how these children are not able to discern the differences between what is safe to consume and what is not.^45,46^

There is limited literature pertaining to CPS reporting practices when a young child tests positive for cannabis, and prior published opinions are mixed.^53,54^ Our study found that encounters with positive cannabis tests had fewer CPS case openings and out-of-home placements than other drug types. Prior literature has shown an overlap of parental illicit drug use and child maltreatment; however, those studies do not isolate cannabis from other illicit drug ingestions and are limited in their ability to provide insight into the association between caregiver cannabis use and child maltreatment.^55,56,57^ There remains a need to establish cannabis-specific recommendations for approaching CPS reporting decision-making. Our study results were mixed with regards to the association of race and ethnicity and insurance status with reporting practices. Prior literature raises concerns of CPS reporting bias related to race and ethnicity and socioeconomic status.^58,59,60,61,62,63,64^ Institutions should standardize their approach to reporting to reduce implicit bias and ensure all families are treated equally regardless of perceived race and socioeconomic status.^65^

Notably in 43.6% of the total medical encounters with positive illicit drugs tests, the child had previously been reported to CPS. Research has demonstrated that prior maltreatment reports are a risk factor for a variety of poor outcomes including injury mortality before age 5, death due to medical causes prior to age 1, and youth suicide.^66,67,68^ The results of our study highlight that there may be opportunities for identification and intervention of caregiver drug use prior to a child ingesting illicit drugs, while raising the question of what services are these children and families receiving.

### Limitations

There are limitations to this study that should also be considered. First, this study is from a single geographic location and may reflect the local reporting practices and laws, thus it is unknown how generalizable the current findings are to other jurisdictions. Second, we assumed that a report made during the encounter window of the positive urine drug test was due to the positive test. Furthermore, there was no definitive way to rule out hospital administration or prescription of drugs that would cause a false positive; however, we did exclude patients who had a prescription in their medical records for opiates, benzodiazepines, barbiturates, or amphetamines to limit this as a potential confounder. Third, it was outside the scope of this study to examine the details of the CPS investigation to identify what other factors may have led to case opening or removal of a child after their urine drug screen was positive. This is an important inquiry for future research. Despite these limitations, this research provides new insight into the medical clinician and CPS responses when a child tests positive for an illicit drug. Further research is needed to understand how to standardize the reporting approach and response to children who are exposed to drugs, especially in light of the widespread legalization of cannabis.

## Conclusions

In this cross-sectional study, fewer than half of children under age 12 who tested positive for illicit drugs during ED and inpatient medical encounters were reported to CPS, and less than 12% were placed in out-of-home care within 30 days. The frequency of children testing positive for drugs increased after the onset of the COVID-19 pandemic. These CPS interactions varied by substance type, suggesting that both reporting parties and CPS workers take the type of substance into account when assessing the need for CPS intervention.

## Notes

Supplement 1. **eTable 1.** Frequency of Most Common Diagnoses for Study Population **eTable 2.** Frequency of Study Population Characteristics by Positive Drug Type

Supplement 2.
Data Sharing Statement
