# Supplementary material for: Child Protection System Interactions for Children With Positive Urine Screens for Illicit Drugs
Source: JAMA Netw Open. 2024 Mar 21;7(3):e243133. doi: 10.1001/jamanetworkopen.2024.3133 (PMC10958236; doi:10.1001/jamanetworkopen.2024.3133)
Supplement: Supplement 1. — eTable 1. Frequency of Most Common Diagnoses for Study Population eTable 2. Frequency of Study Population Characteristics by Positive Drug Type [file jamanetwopen-e243133-s001.pdf]

Supplemental Online Content

Rebbe R, Malicki D, Siddiqi N, Huang JS, Putnam-Hornstein E, Laub N. Child Protection System interactions for children with positive urine screens for illicit drugs. *JAMA Netw Open.* 2024;7(3):e243133. doi:10.1001/jamanetworkopen.2024.3133

eTable 1. Frequency of Most Common Diagnoses for Study Population
eTable 2. Frequency of Study Population Characteristics by Positive Drug Type

This supplemental material has been provided by the authors to give readers additional information about their work.

© 2024 Rebbe R et al. *JAMA Network Open.*

eTable 1. Frequency of Most Common Diagnoses for Study Population

Category	ICD-10 Codes	Frequency, No. (%)
Symptoms, signs, and abnormal clinical and laboratory findings	R00-R99	424 (83.0)
Symptoms and sign involving cognition, perception, emotional state, and behavior	R40-R46	304 (59.5)
Convulsions, not elsewhere classified	R56	72 (14.1)
Abnormalities of heartbeat	R00	67 (13.1)
Drug-related Diagnosis	T36-T39, T4, T5, T65	244 (47.7)
Drug-related diagnosis with unintentional intent	6th character “1”	199 (38.9)
Other Disorders of fluid, electrolyte, and acid-base balance; other and unspecified metabolic disorders	E87, E88	76 (14.9)
Epilepsy and recurrent seizures	G40	56 (11.0)
Injuries	S	53 (10.4)
Respiratory failure, not elsewhere classified	J96	47 (9.2)
Attention-deficit hyperactivity disorders	F90	44 (8.6)
Child Maltreatment	T74, T76	43 (8.4)
Sleep Disorders	G47	19 (3.7)

Notes: categories are not mutually exclusive

© 2024 Rebbe R et al. *JAMA Network Open.*

eTable 2. Frequency of Study Population Characteristics by Positive Drug Type

	Cannabis n = 213	Benzodiazepines n = 139	Amphetamines n = 114	Opiates n = 38	Fentanyl n = 36	Multiple n = 52
Encounter Type						
Emergency Department	119 (55.9)	29 (20.9)	57 (50.0)	16 (42.1)	16 (44.4)	17 (32.7)
Inpatient	104 (48.8)	110 (79.1)	57 (50.0)	22 (57.9)	20 (55.6)	35 (67.3)
Age Category						
< 1 year	<8%[a]	16 (11.5)	24 (21.1)	<30%[a]	[a]	<25%[a]
1-6 years	115 (54.0)	69 (49.6)	37 (32.5)	<30%[a]	25 (69.4)	18 (34.6)
7-12 years	>45%[a]	54 (38.8)	53 (46.5)	20 (52.6)	[a]	24 (46.2)
Child Sex						
Female	111 (52.1)	78 (56.1)	50 (43.9)	21 (55.3)	12 (33.3)	22 (42.3)
Male	102 (47.9)	61 (43.9)	64 (56.1)	17 (44.7)	24 (66.7)	30 (57.7)
Public Health Insurance						
Yes	143 (67.1)	85 (61.2)	79 (69.3)	25 (65.8)	>75%[a]	37 (71.2)
No	70 (32.9)	54 (38.8)	35 (30.7)	13 (34.2)	<25%[a]	15 (28.8)
Child Race/Ethnicity						
Any Race Hispanic	103 (48.4)	76 (54.7)	59 (51.8)	19 (50.0)	16 (44.4)	31 (59.6)
Non-Hispanic White	50 (23.5)	30 (21.2)	39 (34.2)	15 (39.5)	15 (41.7)	13 (25.0)
Non-Hispanic Black	29 (13.6)	12 (8.6)	[a]	[a]	[a]	[a]
After COVID-19 Onset?						
Yes	116 (54.5)	52 (37.4)	40 (35.1)	14 (36.8)	>75%[a]	27 (51.9)
No	97 (45.5)	87 (62.6)	74 (64.9)	24 (63.2)	<25%[a]	25 (48.1)
Prior CPS Report						
Yes	94 (44.1)	45 (32.4)	42 (36.8)	16 (42.1)	21 (53.8)	29 (55.8)
No	119 (55.9)	94 (67.6)	72 (63.2)	22 (57.9)	15 (41.7)	23 (44.2)

Notes: Drug type is not mutually exclusive; [a] masked due to small cell sizes; Race/ethnicity categories other/unknown, Non-Hispanic Asian, and American Indian/Alaska Native are not presented in this table due to small cell sizes, "Other/Unknown" race/ethnicity includes "Other" race and "Decline to Answer".

© 2024 Rebbe R et al. *JAMA Network Open.*
