# Supplementary material for: Child Protection System Interactions for Children With Positive Urine Screens for Illicit Drugs
Source: JAMA Netw Open. 2024 Mar 21;7(3):e243133. doi: 10.1001/jamanetworkopen.2024.3133 (PMC10958236; doi:10.1001/jamanetworkopen.2024.3133)
Supplement: Supplement 2. — Data Sharing Statement [file jamanetwopen-e243133-s002.pdf]

Data Sharing Statement

Data
Data available: No

Additional Information
Explanation for why data not available: The data for this study fall under data sharing agreements that prohibit the re-release of data by the research team.
